# "The Hospital" Nursing Mirror

**Published:** 1901-03-23

**Authors:** 


"The Hospital" Nursing Mirror.

Being the Nursing Section of "The Hospital."

[Contributions for this Section of "The Hospital" should be addressed to the Editor, "The Hospital" Nursing Mirror, 28 & 29 Southampton Street Strand, London, W.C.]

### Notes on News from the Nursing World.

### LORD KITCHENER ASKS FOR MORE NURSES.

Last week we announced that eleven nursing sisters left for South Africa in the s.s. *Briton* on the 9th instant. The Secretary of State for War now informs us that, "in accordance with the telegram of the 12th ultimo from Lord Kitchener, the eleven nursing sisters of the Army Nursing Service Reserve, whose names are subjoined, proceeded to South Africa in the s.s. *Canada* on the 12th instant":—Misses A. M. Breen, L. E. Colston, C. Duncan, S. W. J. Hadden, E. M. Hodgson, J. Murdoch, M. T. Neville, S. M. Paterson, E. Reynolds, S. Smyth, and E. E. Wraxall. Miss Eva Mary Hodgson was trained at University College Hospital and the Royal Hospital for Children at Edinburgh; she has since been staff nurse at the Hospital for Incurables, Oxford, the Royal Alexandra Hospital, Rhyl, and has done private nursing in England and America. Miss Sophia M. Paterson was trained at the Royal Infirmary, Glasgow, and has been staff nurse at the Provincial Hospital, Port Elizabeth, and the Addington Hospital, Durban.

### DEATH OF SISTER O'NEILL.

Great regret has been expressed in Dublin at the news of the death of Miss Ellen O'Neill, of the Army Nursing Service Reserve, which took place on the 12th inst. at the Imperial Yeomanry Hospital at Pretoria, from pleurisy, contracted in the discharge of her duty. Sister O'Neill's illness was mentioned as dangerous in our last issue, but until she was attacked by the disease which, unhappily, ended fatally, she was never absent from duty a single day from sickness. She was a native of Dublin and went out to South Africa from the City of Dublin Nursing Institution at the beginning of the war. The Imperial Yeomanry Committee thoughtfully kept Mrs. Kildare Treacy, lady manager of the Institution, informed as to the course of the illness as the cablegrams arrived, and Lady Howe has telegraphed her kindly sympathy with the family of Sister O'Neill. The Countess Cadogan has also written to Mrs. Treacy a letter of sympathy and condolence.

### THE ROYAL NATIONAL PENSION FUND FOR NURSES.

Probably, all nurses of the Pension Fund are readers of "The Hospital" Nursing Mirror. They will peruse with interest the full report of the annual meeting which we give this week. Here there is only one point which we wish to emphasise. The Chairman brought vividly before the meeting the fact that the Royal National Pension Fund is a co-operative society—at least so far as the policy-holders are concerned. They are assured an equitable distribution of all gain among those who earn it; while the great service on the Council rendered by some of the most influential men in the City of London, with whom time is in a very real sense money, is freely given. These gentlemen, instead of receiving a fee for their advice, consider it an honour and a privilege to assist a movement the primary aim of which is to benefit a class who spend their lives in ministering to the sick. The result of their self-denial is that, apart from office expenses, the society is managed entirely without cost to the policy-holders, thus, as the Chairman pointed out, enhancing the profits of every member who contributes capital to it. This is the true spirit of co-operation as distinguished from the false.

### NURSES AND TOTAL ABSTINENCE.

Alluding to the recent meeting of the Nurses' Total Abstinence League at Stafford House, a correspondent asks what we think of the statement of one of the speakers that "the work of nurses is so hard that they cannot afford to be anything else but abstainers." With every respect for the members of the League who are satisfied that they can best discharge their duties by avoiding stimulants altogether, we are certainly not at all inclined to draw a hard-and-fast line in the matter. That anything beyond the moderate—the very moderate—consumption of alcohol by nurses is bad from every point of view we are convinced. Indeed, it does not require to be argued. But we confess we have no sympathy with the theory that the nurse who totally abstains at all times is therefore likely to do better work than the nurse who drinks a glass of claret or beer at her dinner. Any movement to impose total abstinence as a condition upon those who are engaged in the vocation of nursing deserves just as much condemnation as any attempt to insist that the clergy or the medical profession should take the pledge.

### FOREIGN ATTACKS ON ENGLISH NURSES.

It is not surprising to find that the French and Swiss newspapers, by way of showing their sympathy for the Boers, have been attacking English nurses and the English system of nursing. The complaints made against some of the ladies who endeavoured to play the part of amateur nurses, more especially by Mr. Treves, have been construed as charges against the Army Sisters, and full details of their un-nurselike behaviour have been published in the *Bulletin International* of the Red Cross Societies. In the cause of truth, Dr. Hamilton, of Lausanne, has come forward, and in a recent number of the Society's organ has contributed a clear and concise account of the three classes of nurses who were employed by the English in the South African war, the A.N.S., the A.N.S.R., and the voluntary nurses, and has endeavoured to show that it was against some few of the latter class, the butterflies of Society, that such hard truths had to be said. Excellent though her article is, it will not, we suppose, convince those who wish to consider that everything British is necessarily barbaric, and there will probably be many foreigners who will read with avidity the attack published in the new nurse's paper *Nosokomos* by Sister Karthals, who belongs to the ambulance of the Dutch Red Cross. Her statements that all the Army sisters did "was to settle down to read and do nothing when late in the day they entered the wards, even refusing to give a glass of water themselves, but summoning an orderly," are not worth refuting. Anything English seen through Dutch glasses will, we fear, be the reverse of *couleur de rose* for some time to come, but such accusations do no real harm, and are best treated with the contempt they deserve.

### A LOCAL MEMORIAL OF QUEEN VICTORIA.

It is clear that in the provinces, at any rate, there will be memorials of Queen Victoria of a practical character. At Bridgwater, for instance, it has been determined to further the proposed establishment of a Nurses' Home and Institute as a memorial of the late Soverign. Moreover, a representative committee, consisting of the Mayor and a number of influential local personages, has been formed to carry it into effect. That the idea is popular in the town may be judged by the fact that at the preliminary meeting in the Council Chamber upwards of £80 was promised. A Nurses' Home is badly wanted in Bridgwater, and the inhabitants cannot be blamed for deciding to have their own memorial at their own expense.

### THE FEE QUESTION IN AMERICA.

A QUESTION of great interest to the people of Maine U.S.A., at the present time appears to be the amount of the fee a trained nurse should demand for her services. A combination which has been formed among the nurses, with the approval of the hospital staffs, to maintain a certain figure, is severely condemned by some of the papers. The nurses have, it is stated, determined that they will not go to attend a case for less than 18 dollars a week, and the comment on this is that while the rich can pay the fee, the families who make up the mass of the population in American cities—the carpenters, masons, plumbers, workers in the street and in offices—cannot afford to do so, and that, in fact, the fee asked is more than the head of the household himself often earns as his weekly wage. On the ground that grants are made by the Government to the hospitals, the intervention of the State is suggested, apparently with the idea that the State should regulate the fees of nurses. The fee in this instance is obviously beyond the means of persons with small incomes, but that the attentions of "the State" should be seriously called to the matter, shows how far the principle of State support may be carried when once admitted in regard to hospitals.

### ONE TRAINED NURSE AT STEPNEY WORKHOUSE.

THE case of alleged neglect of a woman in one of the infirm wards at Stepney Workhouse, whose death necessitated an inquest by the East London Coroner, points to the importance of the appointment of a second fully-trained nurse. In addition to the facts elicited at the inquest, our representative has ascertained from the day sister, Miss Owen, that she is the only trained nurse in the wards, and that of the two nurses "who were in attendance, one has had "some experience of similar work," and the other "no hospital training." Naturally enough, after Mrs. Newstead's death, Miss Owen says she now feels that "she hardly dare go to sleep for fear something should go wrong again." But it is intolerable that she should be subject to the tension of being practically always on duty; and the best way of guarding against the repetition of such a deplorable incident as that reported in our columns, is to provide her with a colleague who will be on duty at night.

### BOLTON WORKING MEN AND THE NURSING ASSOCIATION.

AT the annual meeting of the Bolton District Nursing Association it was stated that the total income for last year amounted to £1,026, of which £107 was contributed by the Workmen's Hospital Saturday Committee and £50 by the Co-operative Society. As one of the speakers said, the Bolton working men are very generous, and, in fact, set an admirable example to many of their class. On the other hand, that the patients only subscribed £19, or two per cent, of the working expenses of the institution, is not creditable seeing that many of them were persons who do not support the cause indirectly. We are glad that the Chairman dwelt on the value of voluntary co-operation of the working men's and other organisations as distinguished from the compulsory co-operation of the State or of the Town Council. The feeling that it would be disastrous if nursing associations had to depend for existence upon State or municipal aid is happily increasing rather than diminishing.

### OLDHAM UNION WORKHOUSE.

THE coroner for Oldham has spoken out very strongly respecting the inadequate number of attendants in the imbecile ward at Oldham Workhouse. His remarks were provoked by the evidence given on Friday at an inquest on the body of a man who had been under a certain degree of restraint in the imbecile ward for some days prior to his death. The head attendant stated that there were four attendants by day and three by night for 122 patients, 30 of whom were suffering from general paralysis, and 40 from epilepsy, whereupon the coroner observed that

> It was a very unsatisfactory state of things indeed to have 120 persons more or less insane with only four men to look after them by day and three at night. It was quite obvious to anyone who chose to reflect that it was impossible for so small a number of men to give adequate attention to so large a number of cases. At the last case in which there was an inquest the jury had very properly recommended that additional assistance should be obtained, and he was very sorry that that recommendation—which had been forwarded to the Guardians—had not been attended to.

Why are the Oldham Guardians so negligent in the performance of an obvious duty?

### VILLAGE NURSES IN SCATTERED RURAL DISTRICTS.

THE fourth annual report of the Northumberland County Nursing Association shows that six new districts have been formed since the beginning of 1900; three will employ Queen's nurses, and three "village nurses." Early in the year one district applied for affiliation and was accepted, and a fully-qualified district nurse was obtained for it by the Association. In reference to the training of village nurses, the report says: "The village nurse who is trained by the Association, free of cost to herself, was started with a view to meet the want of trained nursing in scattered rural districts, where it is impossible to raise the sum required to maintain a Queen's nurse. It was also thought that a nurse who could, in addition to her nursing duties, lend a helping hand when necessary in doing the household work, such as cooking, cleaning, &c., would best suit the special needs of the cottagers in these rural districts." As a temporary expedient, the village nurses may be useful, but the aim of all county nursing associations should be to raise sufficient funds to enable them to supply even "scattered rural districts" with fully-trained nurses. The sooner the idea of the nurse who is expected to cook and clean, if necessary, dies out the better, though the fully-trained nurse may often, in an emergency, volunteer to lend a helping hand.

### PREPARING FOR SMALLPOX IN BELFAST.

It is proposed that in view of the possibility of an outbreak of smallpox in the Belfast Infirmary the nurses "who may come in contact with patients should have themselves revaccinated in batches of ten." The matter has been referred to the Infirmary Committee, but the Guardian who made the proposal did not express himself, or is not reported, clearly. We conclude that he did not intend the precaution to be taken at once, but only in the event of a case of smallpox actually occurring in the infirmary. Then it certainly would become necessary, except in regard to nurses who had been very lately revaccinated.

### THE NEW MATRON AT ETON COLLEGE.

Miss Gertrude Ward has been appointed to the post of Matron in College at Eton. She was trained at St. Thomas's Hospital, and was subsequently sister at the English Hospital, Zanzibar, and matron of the British Hospital, Algiers. She has also had considerable experience as a district nurse. It is essential for the matron at Eton to be a trained nurse, as she has charge of the boys when ill. The salary is £200 a year, which is certainly more than most of the salaries in the nursing world.

### THE ST. JOHN NURSING SISTERS.

It is with regret that we learn it has become necessary for the Nursing Sisters of St. John the Divine to contemplate the reduction of the work they have been carrying on for many years in the East End of London. At a recent meeting in Poplar it was stated that lack of sufficient funds, and the steady increase in the cost of living, left them no alternative. The movement was initiated in 1880, when, at the request of the Bishop of Bedford for "help for the East End," the Sisters began their labours there almost single handed, and the people of the district have derived great benefit from it. We hope that in these circumstances the appeal which they have decided to issue for help, will be successful. It has also been most wisely decided to place the position of the Sisterhood before patients, in order that all those who are able to do so should contribute according to their means to the funds of the society. If the patients are generally speaking very poor, some of them at least can afford a trifling contribution. Bearing in mind how much money is raised in Barry, and in some places in the north of England, by payments of a penny a week, we do not see why the experience should not be repeated in connection with the work of the Sisters of St. John.

### PAYMENT FOR SERVICES.

It has been decided by the committee of the Farnborough Diamond Jubilee Nursing Association to make payment for the services of the nurse compulsory on the part of those who can afford it. Hitherto, members needing her ministrations have been "asked" to pay; now they will be "required" to do so. Labourers will be attended free as before, and artisans will be charged 6d., tradesmen 1s., and gentry 2s. The yearly subscriptions which entitle them to her services on these terms are labourers 2s., artisans 3s., tradesmen 5s., and gentry 10s.; non-members who wish to enjoy the advantage are expected to deposit a year's subscription with the nurse according to the class. These are only reasonable proposals, and can scarcely be taken exception to by anyone.

### PROGRESS AT RIPON.

The provision of trained nurses for private patients has not yet been found practicable by the Ripon Victoria Nursing Institution. But the system of the rural nursing of the sick poor has been considerably extended, and the work done in the city by two of the nurses, under the direction of the medical men, has been extremely successful. Instead of the committee having to worry themselves about the financial position, there was a credit balance of £176 at the end of 1900. This shows that the people of Ripon do not merely talk platitudes about the advantages of nursing, but put their hands into their pockets for the purpose of materially assisting the movement.

### NURSES AND THE PAN-AMERICAN EXPOSITION.

In connection with the Pan-American Exposition which opens in Buffalo on May 1st, an emergency hospital has been erected. A number of nurses will be engaged for a month or more, at a nominal salary, board and lodging to be furnished in the Exposition grounds. The reason why the salary will be nominal is that the hours of duty will be arranged so as to give the nurses considerable time to themselves. The superintendent of nurses, who has already been appointed, will be responsible for the selection of nurses, and the regulation of their work, which will be of a somewhat unusual character because of the situation and scope of the hospital. No cases will be kept there longer than is required for their first aid, so that the patients may be moved without danger to their lodging places, or to some other hospital. But a completely equipped operating and dressing-room will wisely be provided for all cases that need prompt surgical aid. Two or more automobile ambulances will be ready to go to any part of the grounds at an instant's notice. Each nurse while on duty will wear the full uniform of the pupil nurse of her training school.

### SHORT ITEMS.

It is proposed to erect a memorial to Prince Christian Victor in the form of a fund for founding and endowing beds in the Princess Christian Cottage Homes for Disabled Soldiers and Sailors. An appeal has been issued commending the movement signed by Lord Roberts, president of the general committee, and Sir Redvers Buller, chairman of the executive committee.—Miss Jessie Hall, the new matron of Portsmouth Workhouse Infirmary, in addition to her experience at Birmingham Infirmary, has for the last five and a half years been superintendent of nurses at the Burnley Union Workhouse Infirmary.—Nursing Sister A. Beith has been discharged from hospital in South Africa to duty.—The next sessional lecture under the auspices of the Royal British Nurses' Association will be delivered at 10 Orchard Street, W., on Thursday next, March 28th, at 5.30 P.M., by the Rev. R. Elliott, on "The Medical Mission Work of the Church Missionary Society."—The s.s. *Simla*, which arrived at Southampton from South Africa last week, had on board Superintendent Sister Drury, A.N.S., and the following members of the A.N.S. Reserve: Sisters E. Edmondson, E. L. Crosser, and R. E. Burnett. Sister E. M. M. Howard was invalided home.—On Wednesday, last week, the nurses of the Staffordshire Institution for Nurses, Stoke-on-Trent, presented the Matron with a handsome travelling bag, the occasion being her birthday. The presentation was accompanied by the expression of a hope that the gift would be useful and serve to remind her of the love and good wishes of all the nurses.

### Medical Lectures to Nurses.

By CARSTAIRS DOUGLAS, M.D., B.Sc., F.R.S. Edin., F.F.P.S. Glasgow, Professor of Medical Jurisprudence, Anderson's College Medical School, and Dispensary Physician, Samaritan Hospital, Glasgow.

### III.—COMA AND ITS CAUSES.

IN our last lecture I just touched on the subject of cerebral hæmorrhage, mentioning that, unlike the other forms of internal hæmorrhage, it is characterised by no external escape of blood, but must be diagnosed by the symptoms it produces, chief among which are coma or insensibility, convulsions, and paralysis. This being so, and remembering that these same symptoms may be caused by other factors than bleeding into or upon the brain, it seems suitable that this lecture should be devoted to a consideration of these important phenomena, and to a brief study of the different conditions under which they arise.

1. *Coma.*—This term is used to designate insensibility caused by some injury, disease, or poison acting primarily on the brain. If a person lose consciousness through loss of blood, or from a shock to the heart, we say he suffers from syncope;" if from interference with respiration, we use the name "asphyxia." Now coma may be due to various totally different causes; and a very great deal depends, both as regards prognosis and treatment, on our ability to state definitely what the origin of the comatose state is. In the first place, as already stated, it may be, and often is, due to bleeding into or upon the brain. This form of coma may come on very suddenly, as in an ordinary severe apoplectic seizure. The insensibility may be very deep, and may never lighten, passing straight on into the sleep of death. The patient lies motionless, with livid and often clammy face, breathing stertorously, and puffing out the cheeks in a curious and typical manner. The face may appear twisted to one side, and examination of the arms and legs will usually reveal paralysis on one side of the body, evidenced by the peculiar lifeless condition of the limbs in question. The nurse should observe the state of the pupils; they are usually moderately dilated, and sometimes unequal. A patient in this state is in a very critical condition. He must be kept very quiet, in a cool darkened room, and moved as little as possible. Nothing should be done, at least at first, save to moisten the lips and tongue with a little water, to attend to the bladder and the bowels under the doctor's directions, and to scrupulously care for the skin lest bed-sores form. Even after sensibility returns and the patient can move and swallow, it must be remembered that at any moment another hæmorrhage may occur. Therefore, do not disturb a patient much who is in this state.

In the next place, loss of consciousness may result from cerebral embolism, or the plugging of an artery in the brain by some material imported from a distant part. This may happen in a case, let us say, of disease of the mitral or aortic valve; a little piece of tissue broken off the valve enters the arterial blood-current, and being swept into the carotid artery, is whirled along till it sticks finally in a vessel in the brain, leading frequently to paralysis and sometimes to coma. Such cases must be treated on general principles—rest, quiet, light diet, attention to skin, bladder, and bowels. Another cause of coma is found in surgical cases where there has been a violent blow on the skull, with perhaps a fracture. All a nurse can do in such cases is to keep the patient as quiet and comfortable as possible until surgical aid arrives.

In a fourth group of cases insensibility supervenes upon an attack of meningitis (usually tuberculous) where there is effusion of inflammatory material within the skull. Such cases are not uncommon, especially in children, but the coma here does not come on suddenly. There is usually a history of serious illness for some days before, with head ache, fever, vomiting, constipation, restlessness, and perhap squint. Such cases almost invariably end fatally, and a nurse can do nothing more than keep the little patient as comfortable as possible. A fifth cause of coma (or at least stupor), also purely a cerebral one, is the ordinary well-marked epileptic fit. After such a seizure the patient may remain in a heavy stupor, almost amounting to coma, for several hours. The history of the case guides one here, and all that is necessary is to let the patient sleep off his heaviness, taking care that he is lying comfortably, breathing properly, and so on.

In the sixth and last class, we may gather together those cases where the coma is due to a poison acting on the brain cells. This class may be subdivided into two smaller sections, according as the poison is generated within the body or is introduced from without. In the first group are to be placed cases of "uræmia," the special form of blood poisoning due to chronic Bright's disease; puerperal eclampsia, which occurs in pregnant or parturient women, and which is also a self-poisoning by noxious bodies retained in the system; and acute yellow atrophy, a rare disease, nearly always fatal, where the liver becomes disorganised and withered. In the second subdivision we have instances of coma due to poisons introduced from without, and in ninety-nine cases out of a hundred the poison is either opium or alcohol. In the former, where the poison is often taken as laudanum, the victim lies deeply narcotised, livid and pale, with a moist skin, breathing slowly, and presenting extremely contracted (pin-point) pupils. In alcoholic poisoning the face is flushed, the pupils moderately dilated, the pulse full, and the breathing heavy and rather stertorous; the breath may smell of spirits.

In all these cases just considered the nurse must simply carry out the medical man's orders, especially in the first-mentioned class; in the second group the proper line of treatment is to evacuate the stomach, to rouse the patient, to administer stimulants in the shape of strong coffee by the mouth or rectum, and strychnine by the skin, and to use the electric current in a suitable manner.

Paralysis and convulsions we shall discuss next week.

### Death in Our Ranks.

LONDON TEMPERANCE HOSPITAL.—Nurses who have been trained at the London Temperance Hospital will hear with regret of the death of Nurse Tabor, who was for fifteen years on the staff of that hospital. Miss Tabor died on Tuesday, March 12th, after a long illness, and was buried at Finchley on Saturday last. The matron and several of the nurses attended the funeral. Nurse Tabor, who was highly esteemed, will be greatly missed by her colleagues.

### Where to Go.

THE CONTINENTAL GALLERY, 157 New Bond Street.—Mr. Westley Manning is showing a charming collection of paintings and drawings entitled "Afloat and Ashore" at these galleries until April 4th.

THE DOWDESWELL GALLERIES, 160 New Bond Street.—A series of pictures in oil entitled "A Year in Wharfedale" by Mr. Rex Vicat Cole are on exhibition at these galleries.

### Royal National Pension Fund for Nurses.

THE Fourteenth Annual Meeting of the members of the Royal National Pension Fund for Nurses was held at River Plate House, Finsbury Circus, London, on Thursday, March 14th, 1901, under the presidency of Sir Henry Burdett, K.C.B. Supporting the Chairman were the following members of council:—Mr. Walter S. M. Burns, Mr. C. Eric Hambro, M.P., the Hon. Egremont J. Mills, Mr. G. Norman, Mr. Edward Rawlings, and Mr. Falconer L. Wallace. Amongst those present were Mr. George King, F.I.A., F.F.A., Mr. Perceval A. Nairne (Chairman of the Seamen's Hospital, Greenwich), Mr. Alfred J. Waley, Miss R. Pritchard (Hon. Secretary of the Benevolent Fund), Miss Shirley (Lady Superintendent Nurses' Institution, Stoke-upon-Trent), Miss Swift, (Matron, Guy's Hospital), Mr. Conrad W. Thies (Royal Free Hospital), Miss D. M. Oldham (Superintendent, St. Katherine's Royal Hospital), Miss E. Baird Johnstone (of the Croydon Nurses' Institute), and several policy holders.

The CHAIRMAN, in opening the proceedings, said: The Secretary will read the notice convening the meeting.

The SECRETARY (Mr. Louis H. M. Dick) having read the notice,

The CHAIRMAN said: Letters of apology have been received from Sir John Watney, Mr. Thomas Bryant, F.R.C.S., Dr. Habershon, Sir William Broadbent, Bart., Mr. Charles W. Trotter, Mr. Frank Capel, Miss Mabel Cave, Miss Monk (King's College Hospital), and Miss E. Fisher (The General Infirmary at Leeds). Ladies and gentlemen, it is now my privilege to propose the adoption of the report. In doing so it gives me great gratification to announce that His Majesty the King has consented to continue to remain the Patron, and Her Majesty the Queen Alexandra has consented to remain the President of the Fund. Those two facts are very important from the point of view of the Council, because with the Queen's death all the offices held by the Prince and Princess of Wales became vacant, and we know that it will be impossible for Their Majesties to do in many other cases what they have consented to do in regard to the offices they held in connection with this Fund. And the importance of that is this, that it is no exaggeration to say that of all the causes which have led to the prosperity of the Royal National Pension Fund for Nurses, not the least of them has been the fact that Their Majesties the King and Queen have been identified with this Fund from the very outset, and actively identified, too. You know that Queen Alexandra has made it her practice periodically to receive the nurses who joined this Fund, and to give them certificates with her own hand—a practice which I hope may be continued in the future. The importance of that is shown in the statistics which the Secretary prepares, for they prove that each year the Queen has held a reception has been a record year, so far as the members who join this Fund are concerned. I am sure that I only express the views of the nurses, as I do those of the Council, when I express our deep sense of indebtedness to and our warm appreciation of the gracious and invaluable support extended to this society by Their Majesties the King, and Queen Alexandra. Before I go into the details I have another duty to perform, that is, to thank the officers of the Fund. I have especially to thank the medical referee, Dr. Potter, and Mr. George King, the consulting actuary. I am quite sure that it is to those two gentlemen, and to their assiduous devotion to the interests of the Fund, that we mainly owe the success of the Sick Pay branch. That branch of insurance is one that most societies avoid because of its difficulties; but the founders of this Fund felt that without sick pay they could not do what it was essential should be done for nurses, who, while spending their lives in looking after the welfare of others, deserve to be looked after by a Fund which can adequately protect them under all circumstances, free from any effort of their own, if they will only show a reasonable measure of thrift. Well, that has been done, and the success of this branch of our work is due to Dr. Potter and to Mr. King; and on behalf of the Council and on your behalf I beg to tender them our sincere and grateful recognition. I have also to thank the Secretary of the Fund, Mr. Dick, and the other members of the staff who give a great amount of time and work to the Society, and do that work exceedingly well, and to the entire satisfaction of the Council. We have also to record our sense of indebtedness to Lady Rothschild, who is President of the Benevolent Fund; to Miss Pritchard, the Hon. Secretary; and to Mrs. Farmer, the Secretary of that Fund, because they have not only distributed the money, which now amounts to about £1,000 a year, with care, but with great discretion—a discretion which has resulted in the whole of the money dealt with being given away without abuse of any kind. I consider that very satisfactory, and I am very glad that Sir William Broadbent alluded especially to his sense of the value of the Junius S. Morgan Benevolent Fund and to the excellent way in which it is administered under Lady Rothschild and the Committee of Hospital Matrons who have charge of it.

### WHAT THE REPORT SHOWS.

I have now to call attention to a few figures in the report. I think it is very satisfactory to know that last year we issued 700 new policies for annuities, and, in addition, 140 policies for sick pay, and that our assets at December 31st, 1900, had increased to £594,000. There is another feature which is a special one of this year, and is very satisfactory—namely, that the number of withdrawals are smaller and show a steady decrease compared with previous years, the tendency on the part of nurses to withdraw from this Fund going steadily down. I consider that if the Council wished for encouragement or wished for a testimony of the importance of this Fund that fact should satisfy any business man respecting the conduct of an enterprise like this. Another feature which is interesting is that the number of pensions which have been paid is steadily going up. At the present time there are 398 pensions which have fallen in, and the amount of money which has been paid away in pensions this year is considerably larger than that of any single year in the previous history of the Fund. The Sickness Assurance branch has increased in popularity. If you remember, we had to make certain regulations in regard to the Sick Assurance branch, which entailed much stricter supervision and re-examination by the medical men, and the nurses did not like this. They thought it was unnecessary; they did not appreciate the reasons for it; and, as a consequence, the number of applications for sick pay steadily fell away. But with the growth of years and the spread of knowledge and understanding among nurses, we have arrived at a time when the number of sick-pay policies issued has again increased, and increased steadily, so that we have this year a very satisfactory record in our Sick Pay branch, and I have no doubt that it will show an upward tendency and yet larger increase in the future. There is one other circumstance I would mention on the report. It is a very important one—I allude to the expenses of management. The expenses of management are only 3·74 per cent. which is below the average and very much below—1¼ per cent. below—the amount which is allowed by the actuary for management expenses on the loading. That means that the amount of profits to be distributed when the quinquennium is completed will be much larger than they would have been if we had higher management expenses. I think you will agree that the results I have mentioned to you are satisfactory as results. They show that this Fund as a business undertaking has attained a stability which will cause it to endure, and that it has won for itself the confidence and support of the people for whose benefit it was established.

### Something Worth Working For.

But there is another matter in connection with this Fund, which is a mutual Fund, which I should like to achieve, and of which I hope evidence will spring up in the near future. We have got the nurses; we have provided them with good security for their investments and a good return upon their money; but we aim at something more than this. We want this greatest of Funds for women workers to have an effect upon the character of the nurses—that is to say, we want to quicken the intelligence of these busy women workers in the world in a business sense, and we want them to become convinced that every precaution which has been taken by the business men who control the management of their Fund was taken in their interest and on the ground of making it more secure, and of benefiting still further those who join it. Now, in order to make this point quite clear, I thought that I might possibly detain you to-day a little while in order to bring to your mind the fact that this is a co-operative society. Now, co-operation in industry means an equitable distribution of all gain among those who earn it. Certainly, so far as the Pension Fund is concerned, the equitable distribution of all gain among the policy-holders is assured, for I would remind you that the Council includes some of the most active and most influential men in the City of London, who give the policy-holders much of their time without fee or reward of any kind. Their service on this Fund as representative men is their expression of the sense of indebtedness which underlies all classes of the community towards those who spend their lives in ministering to the sick; and they regard, as I regard, a position on the Council of this Fund, not only as an honour, but as a privilege, because it affords each of us the opportunity of doing what is far better, in my judgment, than merely giving money—rendering a measure of oneself in the way of personal service. But, from the co-operative point of view, it is very important that all those who join the Society should realise that, as far as the management is concerned, it is absolutely without cost to the policy-holders. Now, in a society for the prosecution in common of a productive enterprise, the profits are shared in accordance with the amount of capital contributed by each member to a co-operative society, and that is what the Royal National Pension Fund for Nurses is. You participate, every one of you, in the profits which are made to the extent you contribute to the Fund, and that is a feature which, if it can once be understood by nurses, will tend to make a great number of those who, from various causes, have not yet awakened to the importance of securing for themselves the protection, shelter, and provision of the Fund look at it in a totally different way, and hasten to join it at the earliest possible moment, because you must remember that in a society of this kind the earlier the age of those who join, the greater are the benefits and the smaller is the cost to themselves individually.

### Sick Pay and Reassurance.

Passing on from the principles of co-operation to their application as illustrated by this Fund, I wish to give you one or two examples. We have, as I have told you, sickness assurance, through which branch £10,000 has already been distributed in sick pay to nurses. That Sick Pay branch could not have been a success and dared not have been undertaken by business men had it not been for the annuity or pension branch of the Fund. Now there you get an instance of the advantages of co-operation. Every nurse who joins this Fund as an annuitant helps to strengthen the Fund, and therefore, indirectly, to benefit those of her sisters who from the circumstances of their work feel that they must obtain for their own protection sick pay as well as a pension. Again, looking at the Fund from the co-operative point of view, I want to illustrate why you should accept the rules and not be restive under them, for what do the rules mean? We have, for instance, strict medical examination and re-examination. Now, if you look at it from the point of view of the individual nurse who, being ill or whose sick-pay policy is expiring and comes to the office wishful to renew her policy, you find that when you say to her, "You must go to the doctor and be re-examined," she is very often indignant, not to say huffy and annoyed. Why is that? Well, she looks upon it that, as she has joined the Fund and been examined once, that should be quite sufficient, and that it is rather fussy and an interference with the liberty of the subject to say to her, "You must go and be examined again." But we are not dealing with the individual nurse under these rules, but with the whole of the policy-holders, because every rule is laid down in order to make this Fund absolutely secure and a certain protection to everyone who joins it; and if you had no rules you would very soon have no Fund. Therefore we have to say to the individual nurse, from the point of view of all the policy-holders, "If you do not choose to be examined, and let your policy fall, you must do so." Now let us look at it from another point of view. You are not the nurse to be re-examined, but the ordinary policy-holder relying in absolute comfort on the security of this Fund, and when you hear of a sister nurse who is ill, or who wishes to renew her sick policy, not being a "good" life, you should know that her re-examination is absolutely necessary in order to protect you, and to protect your security, as one of some 10,000 nurses who have sought the protection of this institution. Therefore if the nurses can only be brought to realise that every rule is made on sound principles for the good and the protection of the whole body of nurses, they will not be annoyed, I hope, and will not misunderstand when, in the ordinary circumstances of business, it is necessary in the case of a nurse who is entering for sick pay to invite her to come up for re-examination by the doctor.

### The Savings Bank Principle.

There is another matter I wish to say something about, and that is the Savings Bank branch, which has an important relation to the co-operative principle. This Savings Bank branch, that is, the right to pay in premiums to this Fund and to put money on deposit, has been organised to meet the special and peculiar circumstances of nurses. So far as an investment on the returnable basis is concerned, it is provided to secure that any nurse who, entering to-day in certain circumstances, and wishing to make a provision for her old age or for the period after her working days are over, can do so with the assured certainty that if before the pension commences her circumstances so change that she will not require her pension, or that the money which she has paid in would in her opinion or that of her friends be of more value to her if she had it in hand, she can come to the office and get her money out with 2½ per cent. compound interest. Speaking last year to you on this very subject, I pointed out that the "plan of withdrawals which we have permitted enables the nurse who joins the Fund to treat it absolutely as a savings bank, because she gets compound interest on her money when she takes it out." Now, we have a letter from a gentleman in the North who writes to call attention to that statement of mine, and who asks, Is it correct? I repeat that it is absolutely correct that the nurse who pays in under the returnable scale will have her premiums accruing at compound interest at $2\frac{1}{2}$ per cent.; but if she withdraws from the Fund she loses the advantage of the co-operative principle, because the Fund was not created and does not exist for the benefit and comfort of those who withdraw from it, but for the benefit and comfort of those who continue to be policy-holders in the Fund. The consequence is that the actuary has advised the Council—and from the outset we have carried out the rule—that if a withdrawal takes place for the private convenience or benefit of an individual nurse, there shall be a deduction of 5 per cent. from the money which the nurse is entitled to receive, in order to protect this Fund from any loss for working expenses incurred in connection with the policy of every nurse who voluntarily retires from it. I hope this point will be clearly understood, that as a matter of business, of protection to the policy-holders, and as a mere act of justice it is absolutely necessary that those nurses who remain permanently associated with the Fund shall have secured to them its full benefits free from any deductions in consequence of the withdrawal of anyone who, having been a member, discontinues to be one. I hope I have made that point quite clear to you and that you understand it.

### How the Benevolent Fund proves Profitable to all.

I have another point to mention in illustration of the principles of co-operation. It is an instance which works rather curiously, but is very satisfactory. We have in connection with this Fund the Junius S. Morgan Benevolent Fund. That Fund was founded in memory of the man, Mr. Junius S. Morgan, without whose encouragement, I may tell you, I do not believe we should have ever had a National Pension Fund at all; and it is a great satisfaction to me to know that that Fund has made so much good and steady progress year by year. Now, in connection with the Pension Fund we have a Loans Department, and any nurse who shows proper cause, who requires a little help or assistance for the purposes of her work or her progress in life at any time, is enabled to come to the Fund, and, on the security of the premiums she has paid in, to borrow up to a certain amount for her own purposes, repaying that money as and when her circumstances—her earnings—allow her to do so. Now, we charge 5 per cent. interest on those loans, and we thought that it would be a good thing that the interest on the nurses' loans should be passed to the credit of the Benevolent Fund. In this way, by means of co-operation indirectly, the nurses who borrow contribute about $\frac{1}{4}$ per cent. extra interest each year, as far as I can make out, to the earning power of all the investments of the Benevolent Branch of the Fund. That, I think, is a very interesting circumstance. It illustrates in a practical way what co-operation means on a large scale, as we have to deal with it in connection with this society, and I am sure that it will be a satisfaction to you, as it is to myself, to realise this fact.

### The Fund as it is To-day.

This Fund has existed for 13 years; that is to say it has been working thirteen years. In those thirteen years upwards of £600,000 have been received and invested, so that before the Fund is twenty years old this organisation of working women should have to its credit at least £1,000,000. There are at the present time issued nearly 10,500 policies. Of these policies upwards of 8,000 have been issued for annuities and 2,500 for sick pay. In other words about 25 per cent.—or one in four—of the nurses who join this Fund enter for sick pay. I should like to say here that I hope that that percentage will tend steadily to increase, because a nurse's work is so full of risks that she never knows what may happen; and in my thirty years' experience of hospital work I have known many cases where very young women, owing to the circumstances under which they do their work, have become permanently crippled and permanently disabled from earning a livelihood. I do therefore take this opportunity to urge upon nurses, as the premiums on the sick policy are a very small addition to what they pay on the annuity policy, that they should look this question of sick pay in the face, and that they should give up, say, the pleasure of buying one bonnet a year, or two or three pairs of gloves, and that they should devote that money so saved to pay the premium necessary to take out a sick policy. In addition to what I have told you about the policies, I may tell you that we are receiving at the present time in premiums from the nurses an income of £74,000 a year. We have paid away in pensions £4,500 a year—that is what we are paying away now—and we have distributed to the sick policy-holders £1,450 during 1900. In addition to this, reverting once more to the Savings Bank branch, and showing you the enormous and striking power of combination, we have actually returned more than £90,000 to nurses who have been obliged to give up their policies from one cause or another. If I was to speak to you for an hour I could not tell you anything more convincing of the necessity of a Fund like this, of its wonderful progress, or produce stronger proofs of the fact that this Fund stands, after thirteen years' work, as the greatest provident Fund for women workers that the world has yet seen.

### The Question of Old-age Pensions.

I have often taken occasion, and I am going, in conclusion, to take occasion once again—as the matter is much in the public mind—to say a word or two about old-age pensions. You have here in this Fund results which no one can question. Those results have been achieved despite the fact that fourteen years ago I was assured by politicians and statesmen, by the heads of the nurses' profession, and by many actuaries that it would be impossible to do what this Fund has accomplished, although Mr. George King stuck to me, like the good man he is, and said, "We have got a very difficult task, but it is not an impossible task, and we are going to do it." And we have done it. It is just exactly the same with respect to this question of old-age pensions throughout this country. We can have old-age pensions to-morrow providing our statesmen will take the trouble really to understand this question, and to make up their minds—both parties and all parties—that they will not approach it except upon a thrift basis. You must have thrift, in my judgment, if you are ever to have an adequate system of old-age pensions for a nation like the English without abuse and without disaster. It can be done in a very simple way, I am confident. If the County Councils and the Guardians were to be federated together for old-age pension purposes it would be possible, from the intimate knowledge possessed by the Guardians in every small community throughout the country of the individual residents, to secure an adequate knowledge of individuals, to devise a just classification of individuals, to encourage independence, and to avoid what must otherwise prove to be ultimately the ruin of England—I mean the destruction of individuality. With a system of old-age pensions founded on thrift, wisely administered under some such organisation as I have indicated—because I do not believe it is necessary to go to the risks or to incur the expense of establishing new machinery —I feel that it is quite as practically possible for England to have an old-age pension system which shall be as beneficial as financially sound, and as widely popular as this Fund has become in regard to one branch, and one branch only, of women workers. And here I would say this, that it is quite a mistake to suppose that the nurse as such, regarding her purely as a woman-worker, is on the whole under any special or peculiarly favourable circumstances for the saving or the earning of money. That is not the case.

Those of us who have most to do with nurses know that, taken altogether, the circumstances under which a nurse has to do and to find her work and has to live place her in the matter of saving certainly in no more favourable condition than many of her sisters in other branches of the labour field. Indeed in some respects she is even less favourably situated than the majority of other women workers. I therefore look forward, and shall continue to look forward, to the establishment of an old-age pension scheme for the United Kingdom which will give satisfaction and comfort to all who require it, and who are entitled to receive it; and when we have attained to this ideal which might easily be attained, then England, I hope and believe, will come to be regarded with more justice as an ideal country; for then, indeed, by her practical common-sense will she have proved that she possesses the true heart of a great Empire. (Cheers.)

I have very much pleasure, ladies and gentlemen, in proposing the adoption of this report, and I have to call upon Mr. Eric Hambro, M.P., to second it. It is a great pleasure to me to think that as the son of his father, Mr. Everard A. Hambro, who was one of the original donors of the £20,000 which enabled this Fund to be established, and who is also the devoted Chairman of our Council, Mr. Eric Hambro should have kindly come down to-day to second the adoption of this report, and that he should also have become a most active and valuable member of the Council of this Fund. All of us older members hope and know that our sons do share our feeling that it is a privilege to be a member of the Council of this Fund, and we hope to carry it on continuously for generations, if necessary, so long as England endures, on a sound and proper basis for the benefit of every woman who has to deal with sickness and who follows the noble calling of a nurse. (Cheers.)

Mr. ERIC HAMBRO, M.P.: Ladies and gentlemen, it gives me great pleasure, I assure you, to second the adoption of this report—a report which shows how satisfactorily the work has been carried on during the past year, and how very popular it has become among the nurses. Sir Henry Burdett, in the able speech which he invariably delivers on these occasions, has gone over the ground very minutely, and explained very clearly the different points connected with the report which you have before you, and therefore I feel that there is very little for me to touch upon which is at all new. Perhaps it would be more in order if I simply formally seconded the adoption of the report, but I feel that in my position, as the son of your Chairman, who is unfortunately absent, I should say something on the general work of the Fund. There is one feature about it which pleases me more than another, and I am sure that it is very gratifying to the rest of the Council—that is, that, for the first time in the past five years the number of sickness policies has increased. I hope that that shows that the nurses have at length come to feel that that scrutiny which they formerly objected to, I believe, was arranged simply to safeguard the interests of those policy-holders whose lives are "good," and to eliminate as far as possible those nurses who, I am afraid, have from time to time, if I may say so, endeavoured to obtain sick pay when, perhaps, they were not in a proper position to receive it. I have heard—though I do not know if it is true—that on one occasion a nurse drew upon us for sick pay to enable her to ride a bicycle. However, I am clear that this branch of the Fund was simply started for those nurses who, either by accident or disease, were incapacitated from attending to their work, and who could come here and obtain the necessary means to enable them to get that rest which they required and deserved. I should like to mention that it seems to me—and it is a fact which is not often dwelt upon—that it is extraordinary that this Fund, started some thirteen years ago, which was founded, as I understand, by one man, should have assumed the enormous proportions it has reached to-day. The fact that it has invested capital amounting to £600,000 speaks very well, I think, for the value and the national importance of the Fund; and I hope that it will continue to prosper and will grow more and more year by year, and that the nurses will realise that it is a Fund in connection with which they can feel, while they are attending to the wants of those who are in sickness or disease, that their old age is being provided for in complete security, because it is absolutely impossible for them in their position in life to look after these matters for themselves. There is another thing that I would point out—and I am not speaking for myself in this matter, for I am the youngest member of the Council—I do not believe that there is a benevolent society or an insurance society that has so many able and influential men in the City looking after its interests as the National Pension Fund for Nurses has.

The CHAIRMAN: Ladies and gentlemen, the report has been moved and seconded; but in putting it to you I should like to express my regret that the Chairman, Mr. Hambro, is not here. I hope, however, that he is more happily engaged in a sunnier clime. He generally goes away at this time of the year, and that is why he does not preside. That is your loss, but let us hope that it is his gain. I beg now to put the motion to you. That the report which has been presented to you be approved, adopted, and circulated in the ordinary way. Carried unanimously.

THE ELECTION OF POLICY-HOLDERS' REPRESENTATIVES.

Two scrutineers of the policyholders' votes—the Hon. Egremont J. Mills and Mr. A. J. Waley—have consented to act. The report of the scrutineers is as follows: "We hereby certify that we have examined the ballot cards received for the voting for the annuitants' and policy-holders' representatives: 1,448 cards were received; 27 were spoiled or contained more than seven names. The following is the result of the voting:—Miss Mabel Cave, 1,345; Miss E. Fisher, 1,346; Miss L. M. Gordon, 1,370; Miss K. H. Monk, 1,356; Miss E. M. Shirley, 1,353; Miss S. A. Swift, 1,346; Miss E. Vincent, 1,332; Miss J. E. Styring, 7; four ladies 2 each, 15 ladies 1 each. We are informed that 4,775 cards were sent out." I therefore declare the first seven ladies duly elected. In accordance with custom, the ballot cards will be destroyed.

Mr. GEORGE KING, F.I.A., F.F.A.: Mr. Chairman, it is with great pleasure that I rise to perform the easy task that has been assigned to me. There is no need for me to say much regarding the work done by the members of the Council. The report which has been before the meeting shows how much that work is, and your own speech shows how the members of the Council attended to it. They are in rather a peculiar position, in that they take no fees like the directors of other companies. Owing to that, the expenses of management of the Fund are kept down very low, with the result that there is available for the assistance of the members so much more than there otherwise would be. The names I have to propose are, I may say, household words among us. There is Mr. Everard Hambro, one of the founders of the Fund, who has been most generous to us with his time and also with his means; Sir William Broadbent, the Hon. Egremont Mills, Mr. Pierpont Morgan, jun. (the name of Morgan is certainly a household word among us), and there is also Mr. Charles Trotter. I beg to move that these gentlemen be re-elected members of the Council. I think that by re-electing them cordially and unanimously we shall be giving them the best vote of thanks that we can possibly give them. We do not expect them to go back to less work, but rather to more work, because the Fund is growing steadily. At the present moment the Annuity Fund alone amounts to £480,000, to which it has risen entirely from the contributions of the nurses, without any adventitious aid. In addition to that, there are the various funds which have been contributed to by benevolent people for the benefit of the nurses. During the past year the Annuity Fund increased by £66,000, and you may fully expect that by the end of the present year it will amount to over £500,000. It has run up rapidly, and all that means more work for the Council. There is one point which interested me very much in the report—the statement that "the class of nurses who imagine that greater advantages are obtainable from other ways of investing their money is gradually disappearing before the class who realise that the benefits accruing from membership of the Fund are considerably greater than those obtainable from non-co-operative societies and from such investments as come within their means." That, I think, is a most interesting statement, and I can bear it out, for this reason—that when questions arose as to whether other more advantageous means of investing the nurses' money existed than those of this Fund I got a good many letters and I made a great many calculations. Of late, I have not been troubled in that way, and I assume that nurses have learnt, what I know is the fact, that this Pension Fund is by far the best medium for their savings. It is very much owing to the great business skill of the gentlemen who so kindly give their time on the Council that the success of the Fund is due. Ladies and gentlemen, I have much pleasure in proposing that the gentlemen whose names I have read out shall be re-elected members of the Council; and I hope—and I am sure—that their re-election will be not only unanimous, but most cordial.

Mr. FALCONER L. WALLACE: I beg to second the motion.

The CHAIRMAN put the resolution to the meeting in the usual way, and, in reading the names again, remarked: "I may say that Mr. Pierpont Morgan is the grandson of Mr. Junius S. Morgan." After declaring the resolution carried, he continued: I have now to move the re-election of Mr. F. Whinney as the auditor. I do so with great pleasure, and, in accordance with the Act, I move that his remuneration be fixed at 35 guineas.

Mr. EDWARD RAWLINGS: I have much pleasure in seconding that.

The CHAIRMAN put the motion, and after declaring it carried, said: We have some nurses present, although only a few, but if there is any question they or anyone present would like to ask I shall be very glad to answer it if I can.

Mr. P. A. NAIRNE: Ladies and gentlemen, I ask you to pass a most cordial vote of thanks to Sir Henry Burdett for presiding to-day. The Royal National Pension Fund for Nurses is really Sir Henry Burdett's monument of his great work in the City of London. It is a monument of industry, a monument of perseverance, a monument of success, and a monument also, I think, of ingenuity in its design, the ingenuity being illustrated and proved by the great success the Fund has attained. I know how cordial is the feeling of nurses towards Sir Henry Burdett for his establishment of this most invaluable Fund to them, and I ask you to express the gratitude which they feel by passing this vote of thanks.

Mr. WALTER S. M. BURNS: Ladies and gentlemen, I have very much pleasure in seconding the resolution. Everyone who knows anything about the National Pension Fund for Nurses must know the immense part that Sir Henry Burdett has taken since its institution in the work of the Fund. We are all, of course, very sorry that our Chairman cannot be with us to-day; but that being so, there cannot be anyone who can fill the chair with more right to do so than Sir Henry Burdett, both for the interest he has taken in the Fund from the start, and for the great service that he has always rendered to it. I have therefore much pleasure in seconding this motion.

The resolution was carried unanimously.

The CHAIRMAN: Ladies and gentlemen, I am very much indebted to you for your vote of thanks, although I need no thanks. The success of the Fund is the best thanks that I can possess. For many years I was in a position of great responsibility over a very large staff of workers among the sick, and I am grateful to have been permitted to organise a means by which all of these workers can, if they wish, make provision for their old age, that provision being absolutely secure. I thank you very much indeed for this vote of thanks, and Mr. Nairne and Mr. Burns especially for their kind words in proposing it.

The proceedings then terminated.

### Appointments.

ALEXANDRA HOSPITAL FOR CHILDREN WITH HIP DISEASE, QUEEN SQUARE, LONDON.—Miss Georgiana E. Spencer has been appointed lady superintendent. She has previously been sister at the Mildmay Nursing Institution, night superintendent at the National Hospital, Queen Square, assistant matron at the Royal South Hants Hospital, Southampton, and lady superintendent at the County General Infirmary, Carlow.

BRISTOL CHILDREN'S HOSPITAL.—Miss S. Mattick has been appointed matron. She was trained at the Bristol General Hospital. She has been sister in the children's ward and in the female surgical wards, and for the past eighteen months she has been night superintendent.

NORTH STAFFORDSHIRE EYE HOSPITAL, HARTSHILL.—Miss H. L. Pearse has been appointed superintendent of nurses, and Miss K. Green has been appointed charge nurse. Miss Pearse was trained at St. Bartholomew's Hospital, and has since been assistant matron and superintendent of nurses at Lambeth Infirmary. Miss Green was trained at the North Stafford Infirmary, Hartshill, and has since been engaged in private nursing at Newtown, Montgomeryshire.

ROTHERHAM HOSPITAL.—Miss H. M. Hickman has been appointed sister of women's and children's ward. She was trained at the London Temperance Hospital, where she has since been sister of the medical ward.

ROYAL ALEXANDER INFIRMARY, PAISLEY.—Miss Laura J. Niven has been appointed sister. She was trained at the Victoria Infirmary, Glasgow, and the Maternity Hospital, Glasgow, and has since been surgical sister, and theatre sister (*pro tem.*).

ROYAL BUCKINGHAMSHIRE HOSPITAL, AYLESBURY.—Miss Eugenie Smith has been appointed charge nurse. She was trained at the General Hospital, Nottingham, and has since been night sister at Oldham Workhouse Infirmary and charge nurse at the Brook Fever Hospital, S.E.

ST. BARTHOLOMEW'S HOSPITAL, ROCHESTER.—Miss Constance MacCarthy has been appointed male surgical and theatre sister. She was trained at the Blackburn and East Lancashire Infirmary for three years. She has since been on the private staff. Miss MacCarthy has also had six months' experience in fever wards.

ST. MARK'S HOSPITAL, CITY ROAD.—Miss Helen Cook has been appointed night sister. She was trained at the London Hospital, and has since been nurse at the Chelsea Hospital for Women and charge nurse at the South-Western Hospital, Stockwell.

### Echoes from the Outside World.

### AN OPEN LETTER TO A HOSPITAL NURSE.

THE "send off" given to the Duke and Duchess of Cornwall and York was as enthusiastic as the general populace could make it. Large numbers assembled in the vicinity of Victoria Station on Friday and showed the goodwill they bore to the travellers by the loudness of their cheers, and I think the Duchess would have been pleased if she could have heard from the lips of homely women and well-dressed ladies in the crowd the remarks of sympathy in the trial she was undergoing in thus leaving her young children behind, and of admiration at the bright looks she managed to maintain. Close to the Green Park a lady cleverly contrived to throw a small bunch of violets and snowdrops into the Royal carriage, saying as she did so, "God speed." With one of those happy inspirations which usually come to us after the event rather than at the time, the Duchess caught the spring blossoms, pressed them to her lips, and bowed and smiled towards the donor. The little tribute and the manner of its reception touched the by-standers at once, and an appreciative cheer went up from all around. On Saturday, the presentation by the King of the decorations and medals of the Royal Victorian Order to officers and men of the Royal yacht *Victoria and Albert* who drew the gun-carriage at Windsor on the occasion of the late Queen's funeral, and who formed the guard of honour at Portsmouth Jetty, was an important part of the ceremony of the day. At the private luncheon given by the Duke and Duchess on board their new temporary home, the King alluded to the pain it gave him to spare his only son just now, but emphasised the fact that he felt, as his mother had done, that it was right the Duke should go. Then, after the last farewells had been said, exactly at four o'clock, the *Ophir* steamed out of the harbour accompanied as far as the Nab lightship by the King and Queen on board the *Victoria and Albert*, and was lost to sight. May she return thus happily and safely to receive a nation's welcome when the summer is ended, and the eight long months shall have passed away!

WARS and rumours of war continue to engage a great deal of attention. In South Africa General Botha, fulfilling the expectations of most people, has declined to accept the terms of peace offered to him by Lord Kitchener, and it again seems as if the struggle with the Boers must be waged to the bitter end. Meanwhile, a most mischievous attempt is being made, I regret to say, by several English papers to produce ill-feeling and stir up strife between this nation and Russia about a dispute in connection with a railway siding at Tientsin, and sensational articles portraying British and Russian soldiers ready to fly at each other's throats have been placarded on several contents bills. Happily, the latest reports tend to show that there is no danger of a trifle terminating in a tragedy. The taste of war which we have had does not make the women of England long for more, whatever it may do in the case of men who are not likely, in any circumstances, to move from the safe shelter of a Fleet Street office.

AT the annual meeting of the British Women's Emigration Association last week, Mr. Chamberlain put in a special plea for funds to be raised which would enable women to go out to South Africa. At the finish of the war, "an end to which they looked forward confidently," he expected that "England would be sending men, some of the most energetic probably of our population, by tens of thousands, by hundreds of thousands," and he was anxious to promote a proportionate emigration of suitable women calculated by their character, by their intelligence, and by their cultivation, to be fitting companions to the men. Mr. Chamberlain's belief that the war is likely to be followed by emigration on a large scale, is shared by many who know South Africa well, and if this should be the case, there is no doubt that there will be almost as great a dearth of women in the new colony as there is of men in the old country. But those who take Mr. Chamberlain's advice as urging women to start off in a very short time will, I fear, find that there is such a thing as being too early in the field. Before the outbreak of hostilities English women in the Orange Free State and the Transvaal frequently found much hostility amongst the Dutch people, and sometimes they were treated with open contempt. If such was the case then, how painfully wider will the breach be now, when there has been of necessity much cause for animosity? That there will be room for some of the surplus women of England in South Africa ultimately is pretty clear, but it must be for those women who are content at present to go on living their existence quietly at home till the proper time comes, when rebellion shall have died down in despair, when the land which has been ravaged by fire and sword shall have begun once more to yield her fruits, and when the Englishmen who have succeeded in living down the prejudices against them, shall be in a condition to protect and look after their fellow countrywomen. Those of my sex who are anxious to emigrate at once should seek some other part of the British Empire as their future home.

IT is extremely nice to have a refrigerator on board the large liners, not only because ice as a luxury for the table or as an aid in illness is very desirable, but also because a cold chamber enables food to be kept sweet for a much longer period than would otherwise be the case. But those who suffered from the disaster on board the American vessel *New York* must almost have wished that the manufacture of artificial ice had never been discovered. An ammonia tank which was in the after part of the vessel, connected with the refrigerating apparatus, suddenly exploded. The released ammonia burst forth in a white cloud, which spread everywhere, overcoming many of the passengers. Those in the steerage were at supper, and made a frenzied rush for the deck, burying their heads in their hands or their arms, in the hope of keeping the fumes from getting into their eyes and down their throats. But several fell as they ran, and at once members of the crew, headed by the captain, came to the rescue. With wet towels tied about their heads, they crept into the cabin and dragged out the victims of the disaster. Many were unconscious for some hours, a large number suffered from inflammation of the eyes and respiratory passages, and two deaths occurred. If the disaster had happened at night the number, of course, would have been vastly augmented.

ELECTRIC tramways have been at work in the suburbs of London for some time, but a public auto-car is still somewhat of a novelty. An attempt, however, is now being made to run a light auto-omnibus, capable of holding eight persons inside and two out, in a district which is much in want of communication. The fares are to be moderate, about the same as those charged by omnibuses generally, whereas the time occupied in the journey will be less than half that which would be required were horses the motive power. Old-fashioned people prophesy that the frequent breakdowns of the apparatus will speedily reduce the pace, and the trial trips were decidedly not without incident and excitement; but the more go-ahead folks are anxious to patronise the scheme largely, especially while it is new, and to ensure its being a success.

### Alleged Neglect at Stepney Workhouse.

By Our Own Correspondent.

An enquiry was held by the East London Coroner on Saturday into the circumstances attending the death of Agnes Newstead in Stepney Workhouse. Two nurses, Rose Biggs and Alice Allen, were called as witnesses. The former was on night duty when the patient died, at 5.40 on Friday morning. She said she had left the ward at 5.30, and was called back in five minutes, when she found the patient was vomiting blood. Further investigation elicited the statement that she did not send for help; that her assistant, Nurse Allen, was with her; that there was no night sister, only the day sister. When the Coroner asked her whether the day sister was not generally sent for in any special case, Rose Biggs replied that it was not a recognised thing. She also said she had not sent for the doctor either; that the patient was too far gone for anything to be done for her; and that she died in five minutes. She had had similar cases, and had always used ice, but in this case she could not get any.

The Master (Mr. Herbert Thomas) said that plenty of ice was kept on the premises, and that the nurse knew how to get it. Alice Allen said she was assistant nurse to the last witness. She admitted having done nothing for the patient in the shape of sending for the doctor, superior nurse, or trying to obtain ice. There were two nurses on duty; there were 14 wards with about 100 patients. Sister Elizabeth Owen was then called. She stated that no report was made to her that the patient had died at 5.40 a.m. At eight o'clock she found the entry in the report book, and that was her first and only intimation. The Master stated that there was a telephone from the dispensary to his bedroom for the purpose of communications being made to him by the nurses in cases of difficulty, and for obtaining anything specially required. Dr. Butler Hogan, medical officer, deposed that no communication was made to him about the deceased's sudden illness, and that the body was removed to the mortuary and the blood washed away without his knowledge. Death was due to hæmoptysis following phthisis. A verdict of "natural death" was returned, and the question of the nurses' conduct was left to the Guardians.

On Monday our representative went to Stepney, and from the Sister Superintendent (Miss Owen), and the Matron (Mrs. Herbert Thomas) obtained full details of the case. Miss Owen is sister superintendent of the infirm wards of Stepney Workhouse, and these are quite separate from the Sick Asylum opposite. She is naturally very much distressed at the occurrence.

In the surgery Miss Owen showed our representative the telephones, one of which communicates with the doctor and the other with the Master's bedroom inside the building. The distance from the ward where the patient was lying to the surgery is very short, only down one flight of stairs. Next Miss Owen led the way to the ward: it has 20 beds, in one of which Mrs. Newstead died. Just outside the door of the ward, and certainly not more than half-a-dozen steps off, is Miss Owen's bedroom.

"I am accustomed to being called," she said. "The statement that it is not a recognised thing to call me is untrue. I am always called for labour cases, all of whom come here, as they do not take them at the Sick Asylum, and I hold the L.O.S. certificate. A slight rap on the door would have wakened me, and I now feel that I hardly dare go to sleep for fear something should go wrong. Again, I am never off duty as long as I am in the building."

Ice, the Matron said, could have been had at once. There was no excuse for the neglect of the nurse in procuring it for Mrs. Newstead.

Our representative having enquired why such an advanced case of phthisis was not received at the Sick Asylum, Miss Owen replied that there was not room there. Acute cases came to the infirmary wards only when they could not be taken in across the way. For her own part, being a hospital-trained nurse of some 10 years' standing, she was glad to have them.

"Are any of the nurses trained?" our representative asked.

"No; I am the only trained nurse here. The Sick Asylum is a recognised training school, but this is not."

"How long have the two nurses referred to been here?"

"Rose Biggs came here last November. She had had some experience of similar work, but I cannot say where. Alice Allen has been here sixteen months. She has had no hospital training."

"Is it a fact that there are 14 wards with about 100 patients?"

"Yes. The wards take 190 cases, but they are rather empty just now, and there are not many serious cases."

"Is it usual for a body to be removed by the nurse without the doctor's knowledge?"

"It would not be allowed in any hospital."

### Everybody's Opinion.

[Correspondence on all subjects is invited, but we cannot in any way be responsible for the opinions expressed by our correspondents. No communication can be entertained if the name and address of the correspondent is not given, as a guarantee of good faith but not necessarily for publication, or unless one side of the paper only is written on.]

### PRESENTATION AT CHELSEA HOSPITAL.

"A Chelsea Infirmary Nurse" writes: Will you kindly allow me to correct an error which appeared in your issue regarding the presentation made to Miss McKay, of Chelsea Infirmary. It is stated that the morocco writing-case, with silver monogram, was given by the matron, Miss Barton. The writing-case, as well as the travelling clock, was the gift of the nurses to Miss KcMay. Miss Barton presented her with a silver pen-holder.

### OVER-WORKED ARMY SISTERS IN SOUTH AFRICA.

"Miss Louise Asman" writes from Connaught Hospital, North Camp, Aldershot, as follows: I read Col. Somerville-Large's letter. I was at No. 6 General Hospital nearly a year before I was, unfortunately, taken sick. I must say I do not think the sisters were in any way over-worked. Certainly we had plenty to do when we first went out, owing to enteric, but not any more than we expected and were quite prepared to do. I consider we were all most kindly and well treated both by Miss Oram, the superintending sister, and our P.M.O., Col. Somerville-Large. My only regret was having to leave No. 6, where I was most happy.

### THE NURSES' TOTAL ABSTINENCE LEAGUE.

"Perplexed" writes: In your brief report of the meeting of the Nurses' Total Abstinence League, no mention is made of the speech of Mr. Pearce Gould, who in strongly urging total abstinence upon nurses, said that the first and foremost of all reasons why they should do so was "that their work is so hard that they cannot afford to be anything else but abstainers." But is this true? As a hard worker myself for a number of years, I have found the occasional use of alcohol helpful, and I know several others whose testimony is the same. I am sure that many nurses would like to have your opinion on this point. Is it quite fair to even suggest that total abstainers are the best nurses?

### For Reading to the Sick.

"AS SILVER IS TRIED."

So stands the Great Refiner, with the glance
Loving yet stern, which pierced th' Apostle's heart,
And reads us through and through. Full well He knows
A soul that He has formed to raise on high
To purest saintliness and, like His own,
"Perfect through suffering." He lays it low,
Deep in the crucible of sharpest pain.
Then gazes down upon it tenderly
Nought seeing there. Yet stay; behold, it comes!
The deep dark shadow of the Cross appears
Upon the suffering soul. The Master's Love
Will be rewarded. Anxiously He strives
With unremitting care to purify
His servant from the faintest earthly stain.
And see! while still He works, the shadow grows
Brighter and brighter yet: the Cross-marked soul
Is glistening as with dewdrops from the Font
Of Baptism. All silvery it shines
That Cross in sunlight—sunlight of His Love!
Yet still a Cross. Then, while the Master bends,
Intently watching with expectant eyes,
A smile of welcome lights His radiant Face,
And lo! the Cross hath vanished, and instead
Appears the likeness of a starry Crown.

*Helen Douglas.*

And if it be that human misery
Is working out God's will, ye suffering ones,
Bear on through all things; for your rich reward
Is greater than our human hearts can grasp,
Is deeper than our finite souls can reach.
Take comfort, the Eternal Will is sweet,
And ye are working out its large behest
Though life is bitter. *F. M. Owen.*

If joy could be brought to sorrow-stricken hearts their path would blossom with good deeds; the gladness within would overflow in acts of heroism and devotion, not uncalled for even yet. And does not joy grow out of sorrow when we see it thus—an infinite and tender joy passing all other? Do we not feel the throbbing of God's heart and see even this sad world beautiful and good beyond conception, beyond hope—and the poor, the miserable, the blighted and shipwrecked lives clothed with sublimity grand, and yet so quietly tender, that pales before it the best joys of earth, fair and blessed though they be?

It is good to be blessed in health, family, friends, in prospects and success; in capacity and power and scope for usefulness; in love returned and growing with its return; giving and receiving more with every year; in deeds of wise beneficence which enrich the lives of nations. It is good to be blessed so; but not so good as to sacrificed, poor, wretched, halt, maimed, and bruised, heart-broken and spiritless, incapable, lost utterly—so sacrificed for man's redemption. That is to be like Christ; it is to hear him say, "Thou drinkest of My cup; with My baptism thou art baptised. I make thee one with Me, the sharer of My joy."

We cry in our agony—we recall the words which fell from the conqueror's lips—"My God, my God, why hast thou forsaken me?" Blackness of darkness and despair and sorrow blotting out God's hand, feebleness sinking without a stay, these, these are not *failure.* In these characters was written the first charter of our deliverance and in these characters it is renewed.—"From "Hinton's Mystery of Pain."

### Notes and Queries.

**The Editor is always willing to answer in this column, without any fee, all reasonable questions, as soon as possible.**
**But the following rules must be carefully observed:—**
**1. Every communication must be accompanied by the name and address of the writer.**
**2. The question must always bear upon nursing, directly or indirectly.**
**If an answer is required by letter a fee of half-a-crown must be enclosed with the note containing the inquiry.**

*Books.*

(286) 1. Will you please tell me of a good book for nurses on fevers? 2. Where can I get the quarterly volume of the "Household (or Family, I am not sure which) Physician?" It is a 7s. 6d. book.—*Ima.*

1. "Fevers and Infectious Diseases: their Nursing and Practical Management," by William Harding, M.D., price 1s.; 2. Nurses can always obtain books of professional topics through the manager of the Scientific Press.

*Home for Aged.*

(287) I want to place an old lady of between seventy and eighty in a home where she would be comfortably maintained for a charge not exceeding 15s. a week.—*C. R. D.*

The Ladies' Home, 53 Abbey Road, St. John's Wood. Apply, Hon. Secretary, 75 St. George's Road, S.W., seems the most suitable.

*Australia.*

(288) Would you kindly tell me if there is any co-operative association for private nurses in either Sydney or Melbourne?—*Colonial.*

Nurses on the staff of the Melbourne Nurses' Home, 6 Parliament Place, take their own fees. Apply for particulars to Lady Superintendent.

*L. O. S.*

(289) Would it be possible to pass the L. O. S. examination without attending lectures? I am a fully-trained nurse and have had three months work in a lying-in ward.—*L. K.*

Candidates for the L. O. S. must satisfy the examiners that they have attended a course of theoretical teaching by lectures or tutorial instruction. As you reside in London why not attend the lectures arranged for nurses at the Midwife's Institute, 12 Buckingham Street, Strand, W.C.

*Young Probationer.*

(290) Kindly tell me what London Hospital would accept me as probationer with salary? I am twenty-two years of age.—*Anxious.*

Perhaps the Lambeth Workhouse Infirmary, Brook Street, Kennington Road, S.E. (Salary first year £7), would accept you.

*Cottage Nursing.*

(291) I have been here three months and have a thorough knowledge of nursing. I should like cottage or district work. Please tell me what training I should require.—*S. J. J.*

You would require at least two years' training, with six months' instruction in district work in order to qualify you as a "Queen's" nurse for district work. It takes three years' hard work to acquire a good knowledge of nursing. The cottage nurses hold a very inferior position.

*Monthly Training.*

(292) I want to learn monthly nursing. Where is the nearest place I can learn, and what should I have to pay?—*E. A.*

There seems to be no public school for maternity nursing near you. It is possible that the matron of the Newark Hospital could inform you of a good private teacher. If not your simplest plan is to come to one of the London lying-in institutions. Fees from £7 5s.

*Maternity Cases.*

(293) I am a certificated monthly nurse. How can I get cases which would enable me to live at home?—*B. C. K.*

Call upon your local medical men, and ask for their support. Ask your friends to recommend you, and give such as are willing to do so a card with your name, address, and terms. Advertise in your local papers.

*Operating Appliances.*

(294) Can you kindly give me the address of firms which make a speciality of fitting up operating theatres?—*E. P.*

You will find several firms advertising in the MIRROR which are in every way excellent.

*Italy.*

(295) Will you kindly tell me how I can get a post as medical lecturer, or as nurse, or as masseuse in Italy. Or a lady with capital to join me in taking a nursing home?—*Masseuse.*

Miss Violetta Fasulo, 7 Via Rondinelli, Florence; and Miss Bryant, Institute for Trained English Nurses, Via Roma, San Remo, Italy, might give you information as to the former; whilst an advertisement might help you as to the latter half of your query.

*Convalescent Home.*

(296) Is it possible to obtain a situation as nurse in a children's convalescent home without previous training or experience. Would a salary be given?—*A. R.*

You can only find a suitable situation by advertising or replying to advertisements.

**Standard Books of Reference.**

**"The Nursing Profession: How and Where to Train." 2s. net; post free 2s. 4d.**
**"The Nurses' Dictionary of Medical Terms." 2s.**
**"Burdett's Series of Nursing Text-Books." 1s. each.**
**"A Handbook for Nurses." (Illustrated.) 5s.**
**"Nursing: Its Theory and Practice." New Edition. 3s. 6d.**
**"Helps in Sickness and to Health." Fifteenth Thousand.**
**"The Physiological Feeding of Infants." 1s.**
**"The Physiological Nursery Chart." 1s.; post free, 1s. 3d.**
**"Hospital Expenditure: The Commissariat." 2s. 6d.**
**All these are published by THE SCIENTIFIC PRESS, Ltd., and may be obtained through any bookseller or direct from the publishers, 28 and 29 Southampton Street, London, W.C.**

